# eGenPub, a text mining system for extending computationally mapped bibliography for UniProt Knowledgebase by capturing centrality

**DOI:** 10.1093/database/bax081

**Published:** 2017-11-13

**Authors:** Ruoyao Ding, Emmanuel Boutet, Damien Lieberherr, Michel Schneider, Michael Tognolli, Cathy H Wu, K Vijay-Shanker, Cecilia N Arighi

**Affiliations:** 1 Department of Computer and Information Science, University of Delaware, Newark, DE 19716, USA; 2 Swiss Institute of Bioinformatics, Centre Medical Universitaire, Geneva, Switzerland; 3 Center for Bioinformatics and Computational Biology, University of Delaware, Newark, DE 19716, USA; 4 Protein Information Resource, University of Delaware, Newark, DE 19716 and Georgetown University, Washington, DC 20007, USA

## Abstract

UniProt Knowledgebase (UniProtKB) is a publicly available database with access to a vast amount of protein sequence and functional information. To widen the scope of the publications associated with a protein entry, UniProt has introduced the computationally mapped additional bibliography section, which includes literature collected from external sources. In this article, we describe a text mining system, eGenPub, which selects articles that are ‘about’ specific proteins and allows automatic identification of additional bibliography for given UniProt protein entries. Focusing on plant proteins initially, eGenPub utilizes a gene normalization tool called pGenN, and a trained support vector machine model, which achieves a precision of 95.3%, to predict whether an article, based on its abstract, should be linked to a given UniProt entry. We have conducted a full-scale PubMed processing using eGenPub for eight common plant species. Altogether, 9025 articles are identified as relevant bibliography for 4752 UniProt entries, among which 5252 are additional papers not in the existing publication section. These newly computationally mapped additional bibliography via eGenPub is being integrated in the UniProt production pipeline, and can be accessed via the UniProtKB protein entry publication view.

## Introduction

UniProt is a comprehensive, high-quality and freely accessible resource of protein sequence and functional information (1). The UniProt Knowledgebase (UniProtKB) is the central database containing information for over 85 million protein sequences (as of Release 2017_05). UniProtKB consists of two sections, one known as UniProtKB/Swiss-Prot (reviewed) that is annotated by experts, and the other, UniProtKB/TrEMBL (unreviewed) that is automatically annotated. Expert curation in UniProtKB/Swiss-Prot includes manual verification of each protein sequence as well as a critical review of experimental data from the literature and predicted data from a range of sequence analysis tools (2). A recent article describes in detail the basis for selection of relevant articles for expert curation (3). Rather than curating all articles about a given protein, UniProt chooses a representative set that maximizes the information content. Thus, an article related to the entry with potential useful information content may not have been included in the entry because: its information overlaps (is redundant) with existing annotations, its main theme is out of scope for UniProt curation, or it is a new publication that has not yet been reviewed by curators. Moreover, UniProt focuses the curation effort on the most widely studied species; therefore, some organisms for which experimental data may be available are not actively annotated.

To complement the curated literature set in UniProtKB/Swiss-Prot with additional publications and to add relevant literature to UniProtKB/TrEMBL entries that have not yet been curated, UniProt compiles additional bibliography from external sources. This additional bibliography consists of literature mapped to UniProt entries from other curated databases (such as, Wormbase (4), Rat Genome Database (5), Intact (6), TAIR (7), GeneRIF (8) and IC4R (9)) added in a collaborative manner. However, this effort provides literature mainly to entries from model organisms, while UniProt entries for other species remain under annotated.

To tackle this issue, UniProt is exploring the use of text mining tools to systematically associate literature to protein entries, with focus on species not yet covered by the curated resources. The detection of protein names in an article by itself is not sufficient for associating the article with the protein entry in UniProt bibliography. For UniProt inclusion, the article is expected to describe at least one experiment conducted on the given protein and in the given species. For example, many articles describe properties of a protein/gene and mention its homologs, e.g. ‘The function of PsBRC1, the pea (*Pisum sativum*) homolog of the maize (*Zea mays*) TEOSINTE BRANCHED1 and the *Arabidopsis* (*Arabidopsis thaliana*) BRANCHED1 (AtBRC1) genes, was investigated’. PMID: 22045922 (10). The above article provides functional characterization of the pea BRC1 protein only, then the article should not be linked to the other homologs mentioned for the purpose of UniProt additional bibliography. Similarly, some gene mentions are listed as background information, e.g. as in the case of soybean LOX1 in ‘It has been known that lipoxygenase (LOX) isozymes exhibit differences in product formation, but most product information to date is for LOX 1 among soybean (Glycine max) LOX isozymes. In this study, LOXs 2 and 3 were purified and used to generate hydroperoxide products in an in vitro system using linoleic acid as a substrate in the presence of either air or O2.’ PMID: 15998134 (11). This article does not appear to contain any experimental study on LOX1 and accordingly, it should not be linked to LOX1. Other times, proteins/genes are part of a methodology, like a marker or a reporter gene, e.g. ‘In this study, the promoter of PtrWRKY89 (ProPtrWRKY89) was isolated and used to drive GUS reporter gene’. PMID: 27019084 (12). This article should not be linked to GUS entry.

In brief, a key requirement for such an association is that the article must describe some experimental data about the protein. It is more important to include a correct article than to miss one. The examples above demonstrate the need to couple the normalization of gene/protein mentions with the concept of ‘aboutness’ to ensure that articles are linked to the relevant UniProt entries. In this article, we describe a method that utilizes a trained support vector machine (SVM) (13) model to predict whether an article, based on its abstract, is appropriate for linking to some UniProt entry as additional bibliography. We use pGenN (14), a normalization tool for plant genes and proteins, for the detection of gene/protein mentions and association to UniProt entries. By utilizing these two tools, we have developed a system, eGenPub that adds articles to the computational mapped bibliography section of UniProt entries. It is currently limited to UniProt entries for eight common plant species: *A.thaliana* (Arabidopsis), *Glycine max* (soybean), *Nicotiana tabacum* (tobacco), *Solanum lycopersicum* (tomato), *Solanum tuberosum* (potato), *Spinacia oleracea* (spinach), *Triticum aestivum* (wheat) and *Zeamays* (maize).

We have evaluated the SVM model for predicting whether the article can be linked to the corresponding UniProt entry. We obtain 95.3% precision using 10-fold cross-validation experiment on a corpus of 450 abstracts. Additionally, we have conducted the full-scale processing of PubMed for the eight common plant species described earlier. Altogether, 9025 UniProt accession–PubMed ID pairs are suggested to UniProt, among which 5252 (3943 of them mapping to reviewed entries and 1309 to unreviewed ones) were not in UniProt previously. The literature collected by eGenPub is integrated in the UniProt production of computationally mapped literature. It is updated every 3 months and can be accessed via the UniProtKB protein entries enhanced view of publications.

## Materials and methods

**Figure 1.**
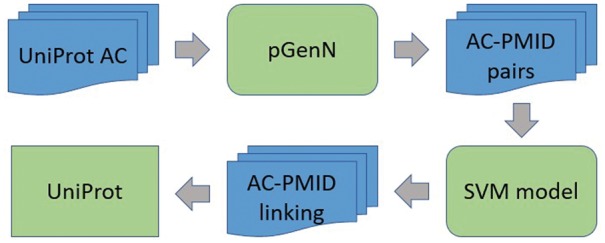
eGenPub system architecture.

eGenPub employs a two-stage process (green boxes in Figure 1). Given a UniProt accession number (AC) as input, it first uses pGenN to search for PubMed abstracts that have mentions linked to that UniProt entry. Then each UniProt AC–PMID pair is converted into a set of features, which are input to the SVM. If a UniProt AC–PMID pair is labeled as ‘relevant’ by the SVM then it means that eGenPub is predicting that article given by the PMID is ‘about’ the protein given by the UniProt AC and therefore, eGenPub predicts that the article can be included in the protein entries bibliography section.

In Figure 1, we describe below a new version of pGenN to obtain the AC–PMID pairs, then we introduce the SVM model for detecting AC–PMID linking, which is the central part of this work.

### Using pGenN to retrieve AC–PMID pairs

pGenN is a state-of-the-art system for plant gene normalization, which automatically detects gene/protein names in the literature and connects them to UniProt AC. It employs three steps: dictionary-based gene mention detection, species assignment and assignment of a UniProt AC. pGenN was previously evaluated and shown to outperform a leading gene normalization tool (14). pGenN obtained an *F*-measure of 88.9% (Precision 90.9 and Recall 87.2%) on a test set that comprised 104 abstracts about plant proteins.

pGenN uses a large plant gene dictionary based on the UniProt protein/gene names. In brief, the dictionary contains proteins and gene names from reviewed entries including recommended name and synonyms from the literature and nomenclature standards, plus locus names and open reading frame (ORFs) names for gene names. It also contains gene names, locus names and ORFs from unreviewed entries, but protein names are used with special consideration. Our previous work (14) showed that the gene names (of the unreviewed entries) were acceptable to add to the dictionary, but the full protein names should be used with caution because they are often very general and would lead to too many non-specific matches (as many of the names arise from direct submissions, propagation of annotation rules developed by UniProt, or other external sources). Since names found in a gene dictionary can also refer to non-gene names and sometimes even common English words, dictionary look-up alone is not sufficient for gene mention detection. pGenN applies a disambiguation process dictionary match. After recognizing the gene mentions, pGenN detects and associates species to those gene mentions. pGenN uses plant community gene naming conventions including the established suffixes used for species (e.g. Zm for maize and Bra for *Brassica rapa*, see table 2 in (14)). The gene mentions are then associated with the species based on a set of rules which extends the rules developed for SR4GN (15). Context of the gene mention and information about the identifiers obtained from the UniProt are utilized to choose one of the candidate identifiers as the final gene normalization result. pGenN also introduced a novel orthographic concept called pivot, which captures the shared part of gene names of same family that helps in all three phases of pGenN.

**Table 2.** List of feature types considered for SVM models

SVM feature	Description
FGE3	Gene is mentioned at least three times
FEQ1	Gene is mentioned once or multiple times
LocTI	Gene is mentioned in title
LocFS	Gene is mentioned in first sentence
LocLS	Gene is mentioned in last sentence
InvCI	Gene co-occurs with an ‘investigation’ word in sentence and within five words
InvSS	Gene co-occurs with an ‘investigation’ word in sentence but beyond five words
SOthT	Another species appears in title
SInT	Species of the gene appear in title
GOth	Another gene is mentioned in title
GOthGE3	Another gene is mentioned at least three times
GFamM	Another gene that belongs to same family is mentioned

For this work, based on the previous evaluation of pGenN (14), a few rules were added primarily to improve the precision of the method. A number of these rules deal with cases where a gene name appears within a longer noun phrase. A few rules, concerned with improving species assignment, decide when a species is not associated with a gene mention, even though they occur close to each other. As an example, because of the new rule Arabidopsis is no longer used to assign to the mention of KCS in the sentence ‘WSL4 is predicted to encode a KCS, a homolog of Arabidopsis CER6’. (16).

pGenN has been run on a large scale and the results are stored so that they can be quickly accessed. For this reason, 527 481 abstracts obtained based on the PubMed query (accessed on 05/2017) ‘plants[MeSH] AND hasabstract[Text]’ were processed by pGenN and the results were stored in a local database called pGenN_DB. The saved results (total of 117 443 PMID–AC pairs) can be searched, sorted and downloaded via a web interface (proteininformationresource.org/pgenn/). Through pGenN_DB, PubMed abstracts that have mentions linked to the input UniProt AC can be retrieved.

### Using SVM for detecting AC–PMID linking

We use SVM to develop a model that suggests when a PMID is an appropriate bibliography entry to add for an UniProt entry. Suppose a gene (normalized to a UniProt AC) occurs in a PubMed abstract (indexed by a PMID). Based on the occurrences of this gene in the abstract, we assign features that are used in the learning of the SVM model. There are a few types of features considered, with additional details described below.

The first type of features is concerned with the frequency of occurrence of the gene in the abstract. Our working hypothesis is that if the gene is mentioned several times in the abstract, it is highly likely that the abstract is ‘about’ the gene. In contrast, if the gene appears only once in the abstract then it is possible that it was just mentioned in passing and the gene is not central to the study reported in the abstract. We have used two features that consider the number of mentions of the gene: feature **‘**FGE3’ counts whether the gene appears at least three times in the abstract and feature ‘FEQ1’ is true/false based on whether the gene is mentioned once/multiple times.

The second set of features is concerned with the location of the mention of the gene in the abstract. Our working hypothesis is that if a gene is central to the study reported in an article, the gene is likely to be mentioned in certain prominent positions of the abstract, e.g. the title, the first sentence of the abstract and the last/concluding sentence of the abstract. Thus, for occurrences of the gene in these positions, we have corresponding features: ‘LocTI’ records whether the gene is mentioned in the title and likewise ‘LocFS’ and ‘LocLS’ keep track of whether the gene is mentioned in the first sentence and last sentence, respectively.

Next, we consider features that are based on whether the gene in question is the object of an investigation described in the article. Table 1 includes a list of lexemes (a lexeme roughly corresponds to a set of forms taken by that single word—e.g. the lexeme ‘detect’ includes the words ‘detect’, ‘detected’, ‘detection …) that indicate an investigation. Although clearly this is not an exhaustive list, it is the current list of words we use to determine if a sentence mentions an investigation. The features that we consider indicate whether a gene appears close to these mentions of investigation. We use the distance between the ‘investigation’ words and the gene to measure the likelihood that the gene is an object being investigated. Based on this notion, we introduce two features ‘InvCl’ (whether the gene is within five words and hence close to the ‘investigation’ word) and ‘InvSS’ (whether the gene co-occurs with an ‘investigation’ word in the sentence but beyond five words of it).

**Table 1.** Lexemes used for ‘investigation’ words

Lexemes			
Analysis	Characterize	Clone	Demonstrate
Detect	Determine	Develop	Express
Investigate	Isolate	Observe	Purify
Result	Sequence	Show	Test

Our working hypothesis is that abstracts that have the above features are likely to be relevant. Conversely, we also considered some features that may indicate when the gene in question is unlikely to be the object of study in the article. The first feature focuses on the species information. Suppose we have normalized a gene mention and we use our model to decide if this gene–PMID pair should be considered as positive. As this gene mention has been normalized, we have already associated a species, say s, to this gene. If this species is not mentioned in the title, but rather some other species is mentioned in the title then we assume it is unlikely the gene–PMID pair is positive. Thus, we consider a feature ‘SOthT’ to indicate that another species name appears in the title that is different from the species of the gene. We also consider a complementary feature ‘SInT’ which is set to true if the species name of the gene in question appears in the title.

Similarly, we considered other features concerning gene names rather than species names. Specifically, we consider a feature ‘GOthT’ that records whether another gene is mentioned in the title and a feature ‘GOthGE3’ that is set to true if another gene appears three or more times in the abstract. These two features could be taken to indicate whether some other gene is object of the reported research. However, we do not preclude the possibility that multiple genes can be studied in the article. We have noticed that when multiple genes are studied in one article they are invariably connected in some way, such as being members of the same gene family. A feature, ‘GFamM’, is introduced to indicate whether any member of the same gene family as the given gene is also mentioned in the abstract. To decide whether multiple genes are from the same gene family, we apply the notion of pivot (14). For example, gene ‘CDK1’ and gene ‘CDK2’ are treated as belonging to the same gene family since they share the same pivot ‘CDK’ but have different suffixes, i.e. ‘1’ and ‘2’. Thus, if multiple genes share the same pivot but have different suffixes, they are treated as from the same gene family.

Table 2 summarizes the type of features described earlier and considered for SVM models.

For the planned application, high precision is preferred over recall. Hence, we considered different feature combinations (Table 3) to see which one might result in high precision. We hypothesized that the feature combinations 1 (Model 1) and 3 (Model 3) (Table 3) might result in high precision and high recall, respectively. Feature combination 2 (Model 2) represents our guess as to what might be a good trade-off between the two metrics. We explored these three combinations by training three different models using SVM-light (17), an implementation of the SVMs, using default parameter settings and a polynomial kernel to learn the models.

**Table 3.** Feature combinations applied on the SVM model

Models	Features
Model 1	FGE3, LocTI, SOthT
Model 2	FGE3, LocTI, SOthT, LocFS, LocLS, InvCI
Model 3	All 12 features

### Evaluation method

We conducted two types of evaluations: an evaluation of the new version of pGenN used for gene normalization, and an evaluation of the SVM model that determines ‘aboutness’. We use the standard measures of precision, recall and *F*-measure for both evaluations.

$$\text{Precision}=\frac{TP}{TP+FP},\;\text{Recall}=\frac{TP}{TP+FN},\;\text{F-measure}=2*\;\frac{precision*recall}{precision+recall}$$

where TP stands for true positive, FP stands for false positive and FN stands for false negative.

We developed a corpus in-house to evaluate the new version of pGenN. The corpus previously used to evaluate pGenN in (14) was not used this time since the new version of pGenN was developed after an analysis of the errors on the first corpus. To build the new corpus, first a set of plant related abstracts were identified by searching PubMed using the query: (‘Proteins’[MeSH] OR ‘Genes’[MeSH]) AND ‘Viridiplantae’[MeSH]. 100 abstracts were selected from the retrieved abstracts, with a selection process that attempted to ensure coverage of the eight common plant species mentioned before: *A.thaliana*, soybean, tobacco, tomato, potato, spinach, wheat and maize. The annotation was completed by five senior biocurators, four from the UniProt plant annotation program (E.B., M.S., M.T. and D.L.) and CNA, all co-authors who did not participate in the development of the system. Altogether 212 instances of AC–PMID pairs were annotated from these 100 abstracts. The evaluation corpus is publicly available at http://research.bioinformatics.udel.edu/iprolink/corpora.php.

As discussed in our previous paper, most of the existing gene normalization tools are either designed for non-plant species or not publicly accessible. Thus, we are only able to compare our results with GNormPlus (17), an updated version of GenNorm (18). Results of GNormPlus were retrieved via PubTator (19). Since GNormPlus normalizes genes to EntrezGene (20) identifiers, we used the ID mapping tool provided by UniProt to convert these identifiers to UniProt ACs.

Another in-house annotated corpus was developed to evaluate the ability of our SVM model to determine the aboutness, i.e. whether the AC–PMID pair should be linked. 450 AC–PMID pairs were selected and marked as either positive (i.e. the pair should be linked) or not. This annotation was completed by one of the authors (C.N.A.), who was not involved in the system development. Altogether, 245 AC–PMID pairs were annotated as ‘positive (for aboutness)’ and the remaining 205 pairs were annotated as ‘negative’**.** The corpus is publicly available at http://research.bioinformatics.udel.edu/iprolink/corpora.php. In our evaluation, we used 10-fold cross validation.

### Pipeline for adding additional bibliography to UniProt entries

The aim of eGenPub is to automatically suggest additional bibliography for the UniProt. Every three months, new plant related abstracts are retrieved from PubMed using the query: (‘Current date’[Date—Publication]: ‘Old date’[Date—Publication]) AND plants[MeSH]. We filter out review articles. eGenPub processes these abstracts and suggests the additional bibliography (in the form of UniProt AC–PMID pairs) to the UniProt consortium. Since the process of adding additional bibliography to UniProt entries has now been automated (with some spot checking), we only want to include those PMIDs that we can be most confident about. Since Model 1 obtains the highest precision among the three models (see ‘Results’ Section), we use it in this automated process.

### Semi-automatic categorization of publications in general annotation topics

To determine the value of the bibliography associated with the entries via eGenPub, we conducted a study where we assigned UniProt entry annotation topics (21) to the suggested publications semi-automatically. For this study, we took a set of UniProt AC–PMID pairs suggested by eGenPub and process these PMIDs using RLIMS-P (22), a tool for extraction of kinase-substrate phosphorylation events. We then checked if any kinase or phosphorylated proteins detected by RLIMS-P mapped to the linked accession. The abstract was tagged with topic for post-translational modification and processing [PTM/Processing] label if the UniProt entry was linked to the substrate, with topic [Function] if it was linked to the kinase, or with both if it was an autophosphorylation event. In addition, all abstracts were tagged by an expert curator for other topics.

## Results and discussion

### Evaluation results of the new version of pGenN

As noted earlier, we conducted an evaluation of the new version of pGenN on an in-house developed evaluation corpus of 100 abstracts, containing 212 instances of AC–PMID pairs. The precision, recall and *F*-measure for both pGenN and GNormPlus can be found in Table 4. Results show that pGenN achieves higher precision and recall. The species assignment component showed significant improvements compared with our previous pGenN version. An error analysis revealed that the majority of the errors were due to gene mention detection issues (19 out of the 26 FNs and 10 out of the 15 FPs), rather than normalization itself. One source of confusion was in the disambiguation between gene names and gene family names. For example, in PMID 26508775 (23), ‘TaPR1’ is recognized as a gene mention, whereas it is a gene family name mention. Finally, only two FPs and two FNs were due to mistakes by the intra-species normalization component.

**Table 4.** Performance of pGenN and GNormPlus on in-house plant corpus

Systems	Precision	Recall	*F*-measure
pGenN	92%	88%	90%
GNormPlus	86%	47%	61%

### Evaluation results of the SVM model

We conducted an evaluation of the ability of the SVM model to correctly predict when a UniProt AC–PMID pair should be linked together. Our evaluation was based on 10-fold cross-validation on a set of 450 pairs.

Table 5 shows the average precision, recall and *F*-measure of 10-fold cross validation using Models 1–3.

**Table 5.** Results of 10-fold cross validation using feature Combinations 1–3

Models	Precision	Recall	*F*-measure
Model 1	95.3%	60.9%	74.3%
Model 2	88.5%	67.8%	76.8%
Model 3	83.2%	77.5%	80.3%

As expected, Model 1 achieved high precision and the lowest recall. The low recall can be mostly attributed to the fact none of the features hold for some pairs. For example, in PMID 7846163 (24), ‘GRF2’ (UniProt AC Q01526) is mentioned only once and none of the features of Model 1 are true. However, the abstract contains a sentence that indicates the gene is subject of the experimental investigation: ‘Two maize (*Zeamays*) genes, designated GRF1 and GRF2, have been isolated and characterized’.

The recall increases with the inclusion of more features (a 7% increase for Model 2, and a 17% increase for Model 3). The previous example, involving the mention of ‘GRF2’ (UniProt AC Q01526) in PMID 7846163, becomes a true positive for Models 2 and 3 as the feature ‘InvCI’ captures the information ‘GRF2’ is an object of an investigation described in that article. However, there is a decrease in the precision for Models 2 and 3, dropping by nearly 7 percentage points and 12 percentage points respectively, compared with Model 1.

### Statistics of full-scale PubMed processing

As previously mentioned, for the purpose of assigning relevant articles to UniProtKB entries, we give more weight to precision than recall. As a result, in our first implementation of the pipeline, we selected the SVM Model 1 to run the full-scale processing applied to the eight common plant species.

Table 6 shows the number of accession (ACs)–abstracts (PMIDs) pairs suggested by eGenPub for the full-scale processing of PubMed. As expected, a subset of these articles is already cited in reviewed entries (second column). However, this constitutes less than half the pairs suggested by eGenPub. More importantly, eGenPub adds additional bibliography to curated entries in Swiss-Prot (which may be source of updates, similar or complementary information, third column), as well as a significant number of not yet curated entries in TrEMBL (adding bibliography for the user to access).

**Table 6.** Statistics of large-scale processing using SVM Model 1

Species	Number of suggested AC–PMID pairs		Number of suggested AC–PMID pairs mapping to		Number of suggested AC–PMID pairs not in UniProt mapping to	
	Suggested	Already in UniProt	Swiss-Prot	TrEMBL	Swiss-Prot	TrEMBL
Arabidopsis	6662	3017	6322	340	3326	319
Maize	588	186	290	298	205	197
Soybean	149	45	45	104	26	78
Tobacco	361	104	142	219	114	143
Tomato	56	15	51	5	38	3
Wheat	369	130	129	240	86	153
Spinach	455	147	136	319	87	221
Potato	385	129	100	285	61	195
Total	9025	3773	7215	1810	3943	1309

The overlapping set of publications in UniProt with those suggested by pGenN is an indication of the relevant bibliography added by eGenPub. To further show the value of the additional papers suggested, we looked at a random set of unique AC–PMID pairs (193 pairs), and mapped them to the general annotation topics in relation to the associated entries. UniProt publications are categorized into the general annotation topics of the entry, namely, Function, Expression, Subcellular location, PTM/Processing, Structure, Sequence, Pathology and Biotech, Family and domains, and Interaction. From these, 150 mapped to a single topic, 35 to two topics and 8 to more than two topics. Table 7 shows the distribution of annotation topics for the additional bibliography suggestions. The results show that the PMIDs added by eGenPub contain valuable information content related to the entry.

**Table 7.** Distribution of UniProt AC–PMID pairs in annotation topics

Topic	Number of AC–PMID pairs
Function	70
Expression	56
PTM/processing	43
Pathology and Biotech	26
Subcellular location	17
Interaction	16
Sequence	11
Structure	5
Family and domain	2

**Figure 2.**
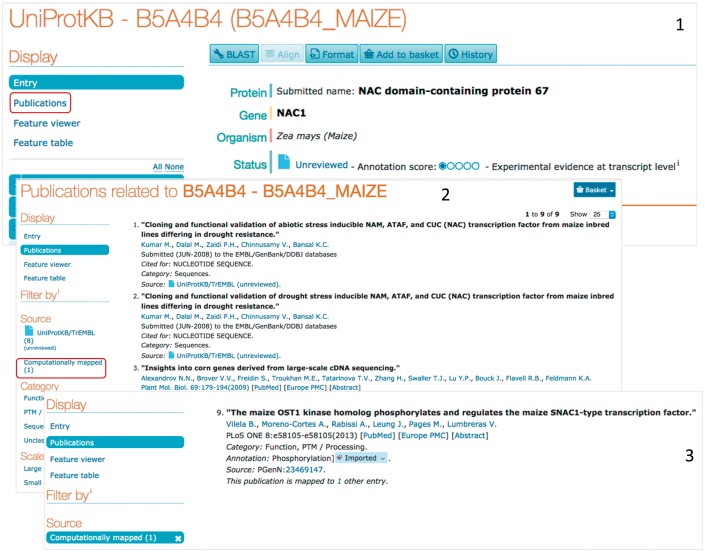
Access to UniProt computationally mapped bibliography. (1) The UniProt entry contains a menu with access to all publications (those in the UniProt entry and those computationally mapped). (2) The publication page can be filtered out based on topics and/or source. (3) Filtering with source: ‘computationally mapped’ display the articles mapped from external resources, including eGenPub.

The bibliography provided by eGenPub is publicly available in the Publication section of the UniProt entry, under ‘computationally mapped’ Section. As an example of the information content added, consider the unreviewed entry B5A4B4, corresponding to gene NAC1 in maize (http://www.uniprot.org/uniprot/B5A4B4, Figure 2). As of release 2017_05, there is no expert annotation on this entry (it is unreviewed), and the automatic annotation information is limited (Figure 2 (1)). The publication section (Figure 2 (2)) lists the source of publications/submissions available. In this case, there are eight submissions listed with no PMIDs in the UniProt entries, and one article in the computationally mapped section. The article added by eGenPub (shown as source: pGenN, Figure 2(3) provides phosphorylation and functional information for Nac1.

## Conclusion

We have presented here a system, eGenPub, to automatically predict whether an article, based on its abstract, is appropriate for additional bibliography for some UniProt entry. The system employs a two-stage process: (i) using a new version of pGenN to detect plant gene/protein mentions in a given abstract and normalize them to UniProt entries, and (ii) utilizing a trained SVM model, to predict whether the abstract should be linked to the normalized UniProt entries. We conducted evaluations on three trained SVM models which use different feature combinations. Results show that Model 1 achieved a precision of 95.3%, and a recall of 60.9%, while Models 2 and 3 achieved lower precision but higher recall. A pipeline has been set up to run a full-scale PubMed processing for the selected eight common plant species using Model 1 to suggest additional bibliography with high confidence. Altogether, 9025 UniProt AC–PMID pairs have been identified, among which 5252 (3943 in UniProtKB/Swiss-Prot entries and 1309 in UniProtKB/TrEMBL entries) were not in the existing UniProt publication section. The additional bibliography suggested by eGenPub is integrated in the UniProt production of computationally mapped literature, and can be accessed via the UniProtKB protein entries view of publications. In the future, we plan to use eGenPub to add additional bibliography for all plant species in UniProt and will provide regular updates in sync with PubMed updates. We will investigate how to improve the recall of eGenPub without significantly affect its precision. Working closely with UniProt, we have demonstrated a robust text mining method for automatically adding bibliography to protein entries in selected plant species, and have shown the added information that these articles bring to the entry. Once integrated into the entries, these additional bibliographies may be used by curators to prioritize and identify entries in need of curation in the future.
